# Coding, stability, and non-spatial inputs in a modular grid-to-place cell model

**DOI:** 10.1186/1471-2202-13-S1-P141

**Published:** 2012-07-16

**Authors:** David Lyttle, Kevin Lin, Jean-Marc Fellous

**Affiliations:** 1 Program in Applied Mathematics, University of Arizona, Tucson, AZ 85721, USA; 2 Department of Mathematics, University of Arizona, Tucson, AZ 85721, USA; 3 Department of Psychology, University of Arizona, Tucson, AZ 85721, USA

## 

Grid cells in the medial entorhinal cortex (mEC) and place cells in the hippocampus are paradigms for population coding of spatial information [1]. Both the spatially-periodic firing fields of grid cells and the spatially localized firing fields of place cells show systematic increases in spatial scale along the dorso-ventral axes of the mEC and hippocampus, respectively [2,3], which has led to speculation that place field size is determined simply by the spatial scale of a place cell’s grid cell inputs. However, in addition to receiving spatially-modulated entorhinal inputs, place cells receive contextual, non-spatial inputs from sources such as the amygdala and hypothalamus [4], which may be important in determining place cell firing properties. These non-spatial inputs are particularly prominent toward the ventral pole of the hippocampus [4], and thus could also play a role in producing dorso-ventral place cell differences.

In order to understand the relative contributions of grid cells and non-spatial inputs in determining place field size and stability, we propose a computational model of the hippocampal-entorhinal network that includes a modular organization of grid cell inputs arranged in order of increasing spatial scale, as is seen experimentally in the mEC. Our underlying place cell model is a firing-rate based model inspired by previous work [5], in which place fields are formed via competition between place cells. We also introduce a dorsoventral gradient in the amount of non-spatial input to place cells, with ventral cells receiving more input from non-spatial sources. Finally, we introduce heterogeneity into the firing rates of grid vertices within individual grid fields. This heterogeneity is observed in experimental recordings [6] but has received relatively little attention in experimental or theoretical work, despite its potential impact on place field stability.

Our main findings suggest that:

1.) For a wide range of parameters, the relative amounts of spatial and non-spatial inputs to place cells plays a more important role in determining place field size and stability than the spatial scale of grid cell inputs. This implies that the dorso-ventral gradient in place field size may reflect a dorso-ventral gradient in non-spatial inputs, rather than grid scale, and is agreement with prior suggestions of a functional distinction between the dorsal and ventral regions of the hippocampus [7].

2.) In our model, place fields are sensitive to changes in the firing rates of the grid vertices of individual grid cells, emphasizing the potential implications of this grid field heterogeneity for place field formation and stability.
